# Sol-Gel Template Synthesis and Characterization of Lu_2_O_3_:Eu^3+^ Nanowire Arrays

**DOI:** 10.3390/mi9110601

**Published:** 2018-11-16

**Authors:** Yahua Hu, Mu Gu, Xiaolin Liu, Juannan Zhang, Shiming Huang, Bo Liu

**Affiliations:** 1 Shanghai Key Laboratory of Special Artificial Microstructure Material & Technology, School of Physics Science and Engineering, Tongji University, Shanghai 200092, China; 79hua@tongji.edu.cn (Y.H.); liuxiaolin@tongji.edu.cn (X.L.); zjn@tongji.edu.cn (J.Z.); smhuang2008@tongji.edu.cn (S.H.); lbo@tongji.edu.cn (B.L.); 2 College of Nanhu, Jiaxing University, Jiaxing 314001, China

**Keywords:** Lu_2_O_3_:Eu^3+^, nanowire arrays, sol-gel method, anodic aluminum oxide (AAO)

## Abstract

Uniform Lu_2_O_3_:Eu^3+^ nanowire arrays were successfully prepared by the sol-gel process using anodic aluminum oxide (AAO) templates. The as-synthesized nanowires are homogeneous, highly ordered, and dense and have a uniform diameter of ~300 nm defined by the AAO templates. The X-ray diffraction and selected area electron diffraction results show that the Lu_2_O_3_:Eu^3+^ nanowires have a polycrystalline cubic structure, and the crystallite size of the Lu_2_O_3_:Eu^3+^ nanowires is confined by the AAO template. The nanowires within the AAO template showed good photoluminescence and X-ray-excited optical luminescence performances for Lu_2_O_3_:Eu^3+^. The emission peaks were attributed to the ^5^D_0_ → ^7^F_J_ transitions of Eu^3+^ (J = 0, 1, 2, 3).

## 1. Introduction

Nanowire arrays have attracted extensive attention because of their fascinating properties, and they can be used in a wide range of areas, including electronics and photonics, energy generation and storage, sensors, and catalysis [1,2,3,4,5]. Over the past decades, many effective approaches have been developed to fabricate nanowire arrays [4,5,6,7]. Among these methods, templated synthesis provides a simple and cost-effective way to precisely control the size, shape, configuration, and direction of otherwise unattainable nanowire arrays. Because of their orderly aligned and well-controllable pores with ideally cylindrical shape, nanoporous anodic aluminum oxide (AAO) has been successfully used as a template to fabricate a vast variety of nanowire arrays using methods such as electrochemical deposition, electroless deposition, chemical and physical vapor deposition, sol-gel deposition, and pressure injection [8,9,10,11]. Sol-gel processing combined with an AAO template has been proven to be a powerful approach for the stoichiometric synthesis of ordered arrays of diverse nanowires because of its advantages such as low synthesis temperature, homogeneous multi-component, and simple equipment [12,13].

Eu-doped Lu_2_O_3_ is an attractive and promising scintillation material because of its significant light yield (20,000 photon/MeV), large effective atomic number (Z_eff_ = 63), high density (9.4 g/cm^3^), and good radiation resistance [14,15]. Moreover, its emission peak at 610 nm, which is attributed to the Eu^3+^ activator, is near the maximum spectral sensitivity of many electronic optical sensors, such as charge-coupled devices, complementary metal-oxide semiconductors, and amorphous silicon flat panels [16,17], all of which make it favorable for being applied to indirect digital X-ray imaging detectors. Columnar-array structured scintillation screens are known to improve the spatial resolution of X-ray imaging because the columnar structure can guide the scintillating light to propagate along the column and can suppress its lateral spreading. Lu_2_O_3_:Eu^3+^ pixel arrays with pixel sizes from 35 × 35 to 80 × 80 μm^2^, which are divided by <10 μm inter-pixel gaps, have been fabricated by laser ablation of Lu_2_O_3_:Eu^3+^ transparent optical ceramics [17]. This process is extremely labor-intensive, expensive, and not commercially viable. Topping et al. developed a cost-effective process to grow columnar coatings of Lu_2_O_3_:Eu^3+^ with a submicron column using chemical vapor deposition [18]; however, the shapes of the columns are irregular, and the thicknesses of the coatings are only about several microns, which is much less than the absorption length of the X-ray commonly used in nondestructive testing or medical imaging.

AAO template synthesis has been used to fabricate scintillation nanowire arrays by both pressure injection and sol-gel deposition [19,20]. The former approach is inapplicable to Lu_2_O_3_:Eu^3+^ because of its high melting temperature (2490 °C). In the sol-gel template method, proper control over hydrolysis, condensation, and deposition is very essential. Though there are numerous reports on the preparation of oxide nanowire arrays by the sol-gel method, the preparation of Lu_2_O_3_:Eu^3+^ nanowire arrays have not yet been reported. The Lu_2_O_3_:Eu^3+^ nanowire arrays as a scintillation screen are good for guiding the scintillation light to propagate along the nanowires and effectively improve the resolution in X-ray imaging. In this study, uniform and dense Lu_2_O_3_:Eu^3+^ nanowire arrays were fabricated by the sol-gel method assisted with porous AAO templates. In addition, the morphology, crystal structure and phase purity, composition, and luminescent properties of Lu_2_O_3_:Eu^3+^ nanowire arrays were studied.

## 2. Materials and Methods

### 2.1. Material

The commercial porous AAO templates (Shanghai Shangmu Tech. Co. Ltd., Shanghai, China) with pores size and length of ~300 nm and 60 μm, respectively, were used in this study. Lutetium nitrate (Lu(NO_3_)_3_·6H_2_O) and europium nitrate (Eu(NO_3_)_3_·6H_2_O) (Shanghai Diyang Industrial Co. Ltd., Shanghai, China) were used as the starting materials. All of the reagents are of analytical grade and used without further purification.

### 2.2. Preparation of Lu_2_O_3_:Eu^3+^ Nanowire Arrays

**Figure 1:**
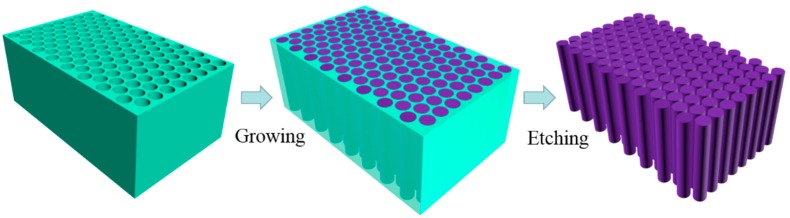
Schematic illustration of the formation of Lu_2_O_3_:Eu^3+^ nanowire arrays.

The main experimental process for the synthesis of Lu_2_O_3_:Eu^3+^ nanowire arrays is shown in Figure 1. Lu(NO_3_)_3_ and Eu(NO_3_)_3_ with an optimum molar ratio of 0.93/0.07 were dissolved in distilled water. The above liquid mixture was stirred at 60 °C in air for the desired length of time until a transparent solution was obtained. The viscosity of the solution was modulated by temperature and time. Then, the porous AAO template was immersed in the above-mentioned solution in a vessel with a water bath at 60 °C. The whole vessel was taken out and sonicated for 15 min every hour to remove the air in the channels of the template. The soaking time was carefully controlled so that the porous AAO could not only be fully filled but also could not be degraded obviously in the nitrate solution. When the sol particle condensed to gel, the AAO template was taken out and sintered in a muffle furnace at 500 °C for 1 h. After carefully cleaning the adhesive crystallized salts on the template surface, the sample was reannealed at 800 °C for 2 h. Finally, the Lu_2_O_3_:Eu^3+^ nanowires were synthesized within the AAO template. Nanopowders were obtained with the residual gel using the same calcination process.

### 2.3. Characterization of Lu_2_O_3_:Eu^3+^ Nanowire Arrays

The morphologies of the samples were characterized using scanning electron microscopy (SEM, Philips XL30, Eindhoven, The Netherlands). For SEM observation of the nanowire arrays, the templates were dissolved by adding a few drops of 2 M NaOH solution. Transmission electron microscopy (TEM) images and selected area electron diffraction (SAED) patterns were measured using a JEOL JEM 2011 transmission electron microscope (Tokyo, Japan) equipped with an energy dispersive X-ray (EDX) spectrometer. Before the TEM observation, partial samples were dissolved with 2 M NaOH solution to obtain the Lu_2_O_3_:Eu^3+^ nanowires. The nanowires were washed with deionized water several times and then dispersed in alcohol. The crystallinity of the samples was determined using a Haoyuan DX2700 X-ray diffraction (XRD, Dandong, China) instrument with Cu K_α_ radiation (λ = 0.154 nm). Photoluminescence (PL) was measured using a Hitachi F7000 fluorescence spectrophotometer (Tokyo, Japan). X-ray excited optical luminescence (XEOL) was obtained using a self-developed X-ray excited spectrometer with a Shanghai Nucl. Med. Instrum. Co. F30-III X-ray tube (W anticathode target, Shanghai, China) set at 50 kV and 2 mA used as an X-ray excitation source. Luminescence spectra were measured using a Zolix SBP300 plate grating monochromator (Zolix Corp., Beijing, China) and a Hamamatsu R928-28 photomultiplier (Hamamatsu, Japan). During the XRD, PL, and XEOL measurements, the Lu_2_O_3_:Eu^3+^ nanowires were packed in AAO templates. All of the specimens were tested at room temperature.

## 3. Results and Discussion

### 3.1. Structure and Surface Morphology of Lu_2_O_3_:Eu^3+^ Nanowire Arrays

**Figure 2:**
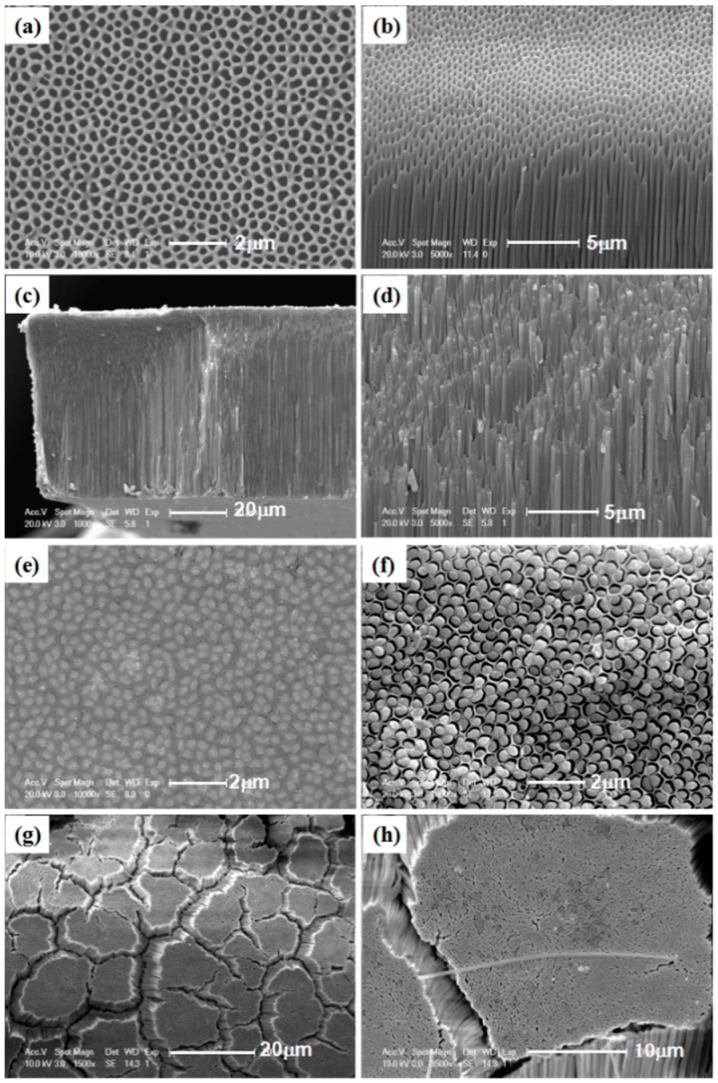
Scanning electron microscopy (SEM) images of the anodic aluminum oxide (AAO) template: (**a**) top view and (**b**) side view; SEM images of nanowires embedded in AAO template: (**c**,**d**) side views of the as-prepared sample at different scales; (**e**) top view of the sample after polishing; (**f**) top view of the sample with the AAO template dissolved for 5 min; (**g**,**h**) top views (at different scales) of the sample with the AAO template dissolved for 1 h.

The morphologies of the AAO template and as-prepared sample were observed by SEM (Figure 2). Figure 2a,b show the SEM images of the AAO template. The average diameter of the pores is ~300 nm and the nanochannels are parallel to each other. Figure 2c,d show the side views of nanowires grown in the AAO template at different scales. The images clearly show that the pores of the AAO template are filled with nanowires to form a monolithic block, and the nanowires have a regularly arranged structure along the AAO nanochannels. The top morphology of the sample after polishing is shown in Figure 2e, which clearly indicates that the nanowires have a uniform diameter of ~300 nm, corresponding to the pore diameters of the template. Figure 2f shows the top view of the sample with the AAO template dissolved for 5 min. Figure 2g,h show the morphology of the sample with the AAO template partially dissolved for 1 h at different scales. The nanowires were isolated from the template to form many clusters. They tend to be parallel to each other, orderly, and perpendicular to the template surface. It is interesting that a single nanowire with the length of about 30 μm jumped on the top the nanowire clusters, as shown in Figure 2h. Although its length is less than the thickness of the AAO template because of a fracture during the operation, the nanowire looks perfect.

**Figure 3:**
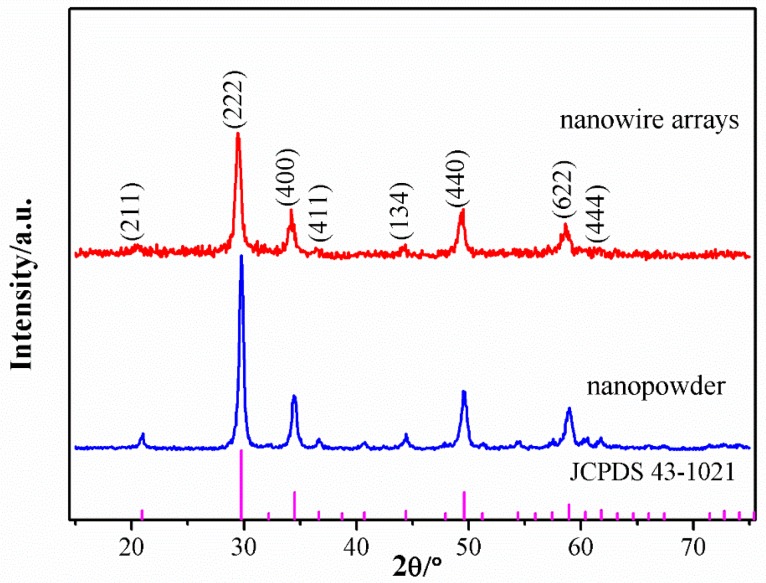
X-ray diffraction (XRD) patterns of the prepared nanowire array within the AAO template and nanopowder.

The crystallinity of the nanowire array within the AAO template and that of the nanopowder annealed in the same conditions were characterized using XRD. In Figure 3, all of the main diffraction peaks of the nanowire array and nanopowder were indexed to the cubic structured Lu_2_O_3_ (JCPDS card No. 43-1021); however, the diffraction peaks of the nanowire array, which is in the background of the Al_2_O_3_ template, are lower and wider than those of the nanopowder. The average grain sizes of the Lu_2_O_3_ nanowires and nanopowder calculated by Scherrer’s equation are 18.3 nm and 26.0 nm, respectively, although the nanowires and nanopowder were synthesized by the same sol-gel and sintering process; this indicates that the coalescence of Lu_2_O_3_ nanocrystallites is confined by the AAO nanochannels [20].

**Figure 4:**
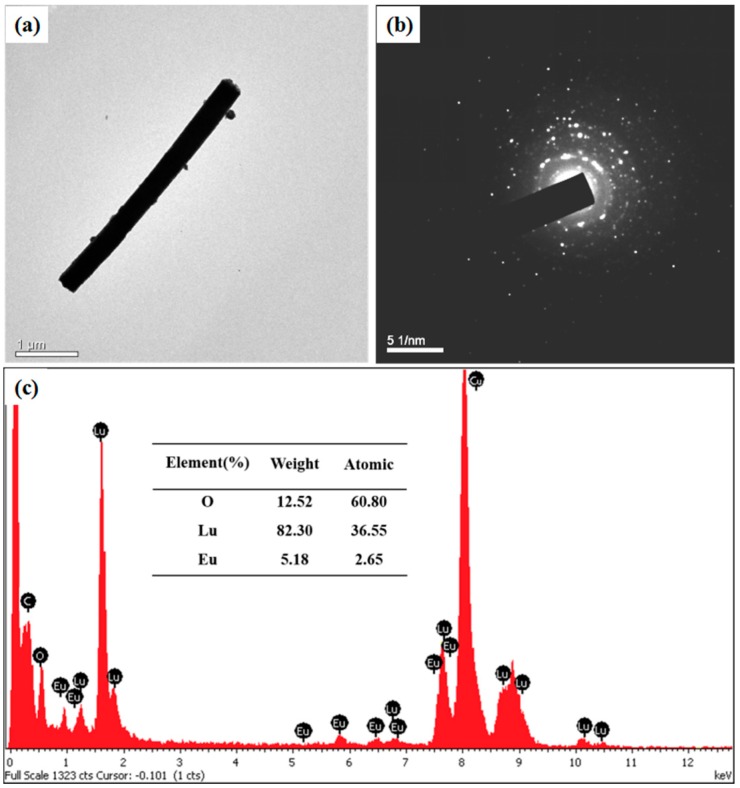
(**a**) Transmission electron microscopy (TEM) image; (**b**) selected area electron diffraction (SAED) pattern; and (**c**) EDX sprectrum of a Lu_2_O_3_:Eu^3+^ nanowire.

Figure 4a shows a typical TEM image of a single Lu_2_O_3_:Eu^3+^ nanowire. Notably, the Lu_2_O_3_:Eu^3+^ nanowire is dense and uniform with a diameter of ~300 nm. The SAED pattern of the Lu_2_O_3_:Eu^3+^ nanowire, shown in Figure 4b, verifies its polycrystalline structure. The result can be confirmed by the polycrystalline XRD pattern of the nanowire illustrated in Figure 3. The elemental composition of the nanowires was measured using EDX spectroscopy. Figure 4c shows the EDX spectrum of the Lu_2_O_3_:Eu^3+^ nanowire. The element C and Cu come from the carbon-coated copper wire mesh in the TEM experiment. The molar percentages of Lu, Eu, and O elements in the nanowires are shown in the inset table of Figure 4c. The molar ratio of Lu and Eu to O is 2.0:3.1, indicating that the nanowires are only made of Lu_2_O_3_:Eu^3+^. Moreover, the molar ratio of Lu to Eu is 0.93:0.07, which is in agreement with the stoichiometric ratio of the raw materials.

### 3.2. Luminescence Properties of the Lu_2_O_3_:Eu^3+^ Nanowire Arrays

**Figure 5:**
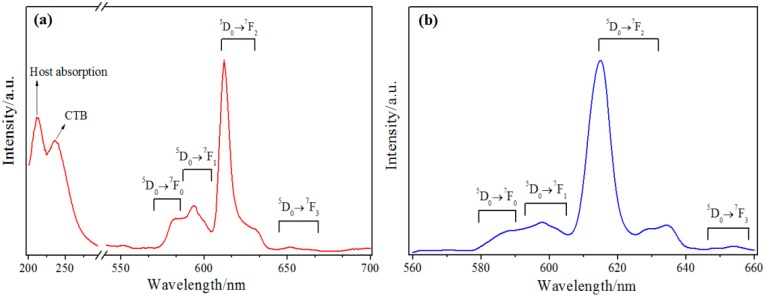
(**a**) Excitation (λ_em_ = 612 nm) and emission (λ_ex_ = 254 nm) spectra and (**b**) X-ray excited optical luminescence spectrum of a Lu_2_O_3_:Eu^3+^ nanowire array within the AAO template.

The excitation (λ_em_ = 612 nm) and emission (λ_ex_ = 254 nm) spectra of a Lu_2_O_3_:Eu^3+^ nanowire array within the AAO template are shown in Figure 5a. The excitation spectrum has two bands with maxima at 212 nm and 236 nm, and these are related to the well-known host absorption of Lu_2_O_3_ and the charge-transfer (CT) transition from O^2−^ to Eu^3+^, respectively [21]. The emission spectrum exhibits a strong peak with a maximum at λ_max_ = 612 nm, which corresponds to the ^5^D_0_ → ^7^F_2_ transition of Eu^3+^. The other weak peaks can be ascribed to the ^5^D_0_ → ^7^F_J_ transition of Eu^3+^ (J = 0, 1, 3). These spectral data indicate that the nanowires within the AAO template show good photoluminescence performance for Lu_2_O_3_:Eu^3+^. The XEOL result of a Lu_2_O_3_:Eu^3+^ nanowire array embedded in the AAO templates is shown in Figure 5b. The spectrum is very similar to the one excited by ultraviolet (UV) light. The emission peaks can also be assigned to the ^5^D_0_ → ^7^F_J_ transition of Eu^3+^ (J = 0, 1, 2, 3). The result indicates that the Lu_2_O_3_:Eu^3+^ nanowire arrays within the AAO templates can be applied in high-resolution indirect digital X-ray imaging detectors.

## 4. Conclusions

Lu_2_O_3_:Eu^3+^ nanowire arrays were successfully prepared by the sol-gel process assisted with AAO templates. SEM images of the sample show that the nanowires grew into the nanochannels of the AAO template and are uniform, highly ordered, and dense. A TEM image of a typical nanowire shows that it is homogeneous, with a diameter of ~300 nm as defined by the porous AAO template. XRD, SAED, and EDX results reveal that the nanowires are composed of polycrystalline cubic Lu_2_O_3_:Eu^3+^. The average crystallite size of the Lu_2_O_3_:Eu^3+^ nanowires is smaller than that of nanopowder prepared by the same sol-gel and sintering process, because of the confinement effect of the AAO template. Both PL and XEOL spectra show that the nanowire array within the AAO template has good luminescence performance for Lu_2_O_3_:Eu^3+^. The emission peaks can be ascribed to the ^5^D_0_ → ^7^F_J_ transitions of Eu^3+^ (J = 0, 1, 2, 3). The results indicate that the Lu_2_O_3_:Eu^3+^ nanowire arrays as scintillation screens can be produced using this cost-effective technique. Such scintillation screens are expected to improve the spatial resolution of X-ray imaging.
